# Birth Weight and Childhood Psychopathology in the ABCD Cohort: Association is Strongest for Attention Problems and is Moderated by Sex

**DOI:** 10.1007/s10802-021-00859-0

**Published:** 2022-01-24

**Authors:** Niamh Dooley, Mary Clarke, David Cotter, Mary Cannon

**Affiliations:** 1 Department of Psychiatry, Royal College of Surgeons in Ireland, Dublin, Ireland; 2 Trinity College Institute of Neuroscience, Trinity College Dublin, Dublin, Ireland; 3 Department of Psychology, Royal College of Surgeons in Ireland, Dublin, Ireland; 4 Department of Psychiatry, Beaumont Hospital, Dublin, Ireland

**Keywords:** Birth weight, Foetal growth, Gestation, Child mental health, Attention, ADHD

## Abstract

Many studies have shown low birth weight is associated with psychopathology later in life, particularly attention-deficit/hyperactivity disorder (ADHD). The association is well-replicated, independent from a variety of potential familial confounds, and follows a dose–response curve (decreasing birth weight linked with increasing odds of disorder). However, the specificity of the association to attention problems is called into question by the extent of comorbidity in ADHD, and recent findings that the association is stronger for autism than ADHD. We test the relative dose–response strength of birth weight on multiple aspects of behavior to explore specificity of the effect to attention problems. We also test recent suggestions that the association between birth weight and attention problems is driven by males. Our sample consisted of 9,076 children aged 9–10 from the United States (Adolescent Brain Cognitive Development study). Outcomes included 9 problem-scales and the total problems scale from the Child Behavior Checklist (CBCL). Attention problems were the most strongly associated with birth weight after controlling for gestational age, potential familial confounds, and multiple testing, supporting the outcome-specificity of this association. Contrary to recent registry-based findings, an association between birth weight and an autism scale was not observed. Sex moderated the effect of birth weight on total problems, attention problems and aggressive behavior such that these inverse associations were strongly driven by males. Our findings have strong implications for sex-specific prediction and etiological models of childhood psychopathology.

**Supplementary Information:**

The online version contains supplementary material available at 10.1007/s10802-021-00859-0.

## Introduction

### Birth Weight and ADHD

Attention-deficit/hyperactivity disorder (ADHD) and its symptoms have been the most extensively studied psychological effects of low birth weight. A recent meta-analysis of 88 independent studies (Momany et al., 2018) reported that birth weight had a small but significant effect on ADHD symptoms (r = -0.15). Four studies found that identical twins with significantly different birth weights had differing susceptibilities to ADHD, such that the lower birth weight twin was more likely to have symptoms (Ficks et al., 2013; Groen-Blokhuis et al., 2011; Hultman et al., 2007; Pettersson et al., 2015), suggesting the effect is independent from the genetic and environmental factors shared by twins (including approximate gestational age). Birth weight discrepancies amongst (non-identical) siblings also predict the difference in odds of ADHD diagnosis (Class et al., 2014; Pettersson et al., 2019). The association between lower birth weight and ADHD is therefore well-replicated, independent from familial confounds and has plausible biological mechanisms (e.g. ischemia-hypoxia, T. F. Smith et al., 2016).

### Birth Weight as a Continuous Risk Factor

The association between lower birth weights and risk for ADHD appears to be robust, yet the transition from observation to prediction at the individual level has been hampered by the historic preference for case–control studies. Many studies have taken the binary approach to birth weight (e.g. under 2.5 kg or weight-for-gesational-age below the 10^th^ percentile), showing an increased risk of psychopathology amongst these groups (meta-analyses: Aarnoudse-Moens et al., 2009; De Mola et al., 2014; Mathewson et al., 2017) however it is clear from studies using continuous measures of birth weight (Abel et al., 2010; Pettersson et al., 2015, 2019; Wiles et al., 2006) that the risk is best described as continuous. To generate useful prediction models in psychiatry, such as those that already exist in other areas of medicine (e.g. heart disease), it would be first helpful to accurately model the continuous dose–response association between birth weight and psychological outcomes. To do so, we need to verify which outcomes are relevant (i.e. specificity) and moderating factors (e.g. sex) that may alter the slope or shape of the association.

### Specificity to Attention Problems

Are the psychological effects of low birth weight specific to attention problems? “Specificity” of an association is one of the nine criteria for causality proposed by Hill (1965) and may improve our aetiological understanding of how birth weight relates to ADHD symptoms, also aiding transition from population statistics to individual-level prediction. The specificity of the association between birth weight and ADHD is brought into question by high numbers of concurrent and sequential comorbidity among those with any mental health issues (e.g. Caspi & Moffitt, 2018). For instance, over half of children and adolescents with ADHD have at least one other comorbid psychiatric disorder (Jensen & Steinhausen, 2015; Yoshimasu et al., 2012) and there is considerable overlap between the inherited/genetic basis of ADHD and other disorders including autism, depression, bipolar disorder and schizophrenia (Selzam et al., 2018; Cross-Disorder Group of the Psychiatric Genomics Consortium, 2013). Further, there is some evidence that birth weight is non-specific risk to multiple distinct psychopathologies including affective disorders, schizophrenia, substance-use disoders and stress-related/somataform disorders (Abel et al., 2010; Burnett et al., 2011; Davies et al., 2020; De Mola et al., 2014). This evidence calls into question whether the effects of birth weight are truly specific to ADHD symptoms rather than general psychopathology.

The studies that suggest birth weight is a continuous risk factor that is specific to ADHD, are limited by their study designs. Ficks et al. (2013) and Momany et al. (2017) compared the strength of the birth weight effect across ADHD symptom scales (inattention, hyperactivity/impulsivity) and symptoms of two externalizing conditions: conduct disorder and oppositional disorder. Both found that inattention was most strongly associated with birth weight, an observation which remained true in a sub-sample of paired-twins (Ficks et al., 2013), however interpretation of specificity from these studies is limited by the small number of comparitive outcomes (externalizing only). Two other studies (Class et al., 2014; Pettersson et al., 2019) used the Swedish psychiatric registries to compare birth weight and psychiatric diagnoses from centralized medical records on the entire Swedish population and in doing so captured a wider variety of mental disorders for a large sample. Both of these studies used a paired-sibling design, thus controlling for shared family environment. Pettersson et al. included 11 psychiatric disorders and found that lower birth weight was only significantly associated with autism, ADHD, OCD and depression (28%, 14%, 7%, 5% increased odds, respectively). Similarly, Class et al. included 5 psychiatric outcomes and found the strongest inverse association between birth weight and autism (B = -0.07; from within-sibling model), followed by ADHD (B = -0.04) and psychotic/bipolar disorders (B = -0.02). Both these registry studies suggest that autism is most reliably linked with decreasing birth weight, with roughly double the effect size of ADHD. This raises the question whether effects of birth weight are generally neurodevelopmental (influencing the odds of both autism and ADHD) and whether the historical focus of research in this field on ADHD has been misplaced.

In this study, we test the specificity of the psychological effects of birth weight to attention and other neurodevelopmental problems as opposed to general psychopathology.

### Sex Differences

In studies of birth weight and subsequent mental health, sex is often controlled for as an additive covariate. Birth weight is therefore assumed to relate to outcomes in an equivalent manner for males and females but Momany et al. (2017) found, in sample of over 700 children, that sex moderated the relationship between birth weight and ADHD symptoms (inattention, hyperactivity) such that the negative association was driven by boys. Reviews suggest males born preterm and/or low birth weight are at higher risk than females of neurocognitive problems (DiPietro & Voegtline, 2017) and even mortality (Vu et al., 2018) thereby providing plausible basis for the male vulnerability. However, Pettersson et al. (2019) found no sex difference in the effect of birth weight on odds of mental illness and Murray et al. (2015) found the opposite, that being born SGA increased childhood attention problems in females but not males. We address these inconsistencies by testing whether sex moderates the associations between birth weight and a range of mental health outcomes.

### Gestational Age vs Foetal Growth

A poor or slow foetal growth rate is represented by a foetus being underweight compared to population norms. Examples of this are being small-for-gestational-age (SGA; birth weight below the 10^th^ percentile for their sex and gestational age) or experiencing intra-uterine growth restriction (IUGR). Low birth weight can result from early gestational age, slow foetal growth rate, or both. While early gestational age and reduced foetal growth share some risk markers/factors (maternal infection, maternal smoking, preeclampsia etc.) it is important to parse their respective contribution to effects of birth weight as they remain distinct biological paths with partially unique risks factors (Heaman et al., 2013; Lang et al., 1996).

The effect of birth weight on the risk of attention problems appears to be best explained by foetal growth rather than gestational age, an observation supported by twin studies (Ficks et al., 2013; Groen-Blokhuis et al., 2011; Hultman et al., 2007; Pettersson et al., 2015), by the effect of birth weight remaining within term-born children (Groen-Blokhuis et al., 2011; Pettersson et al., 2015), and by meta-analytic evidence showing that gestational age does not contribute significantly to heterogeneity of the effect of birth weight (Momany et al., 2018). However, the contribution of gestational age Vs foetal growth to birth weight effects on other aspects of mental health is less clear. We therefore explore the relative contribution of foetal growth Vs gestational age to various scales of mental health.

### Aims

The current study explored the association between birth weight (measured continuously) and childhood mental health in a large population-based sample of 9–10-year-old children from the United States (Adolescent Brain Cognitive Development Study; ABCD). We aimed to investigate: (1) the specificity of the association of birth weight across 10 different outcome scales generated from the CBCL (Child Behavior Checklist) including an attention problems, autism spectrum disorder (ASD) and total problems scale; (2) whether any of these associations were moderated by sex; (3) which associations were better explained by gestational age Vs foetal growth.

## Methods

### Data Source

This was a secondary analysis of data from the Adolescent Brain Cognitive Development (ABCD) study, a prospective cohort study of children, aged 9–10 at baseline and recruited from 22 study sites across the United States. Children were born between 2006 and 2008. The data used in this study were drawn from ABCD Release 3.0 and can be found via our registered NDA study page (https://doi.org/10.15154/1520466).

The 22 geographic locations that comprise the ABCD research sites are nationally distributed and generally represent the range of demographic and socio-economic diversity of the United States. Within study sites, consenting parents and assenting children were primarily recruited through a probability sample of public and private schools as well as summer camp programs and community volunteers. Further detail on the sample design and procedures employed in the recruitment of the baseline sample are described in Garavan et al. (2018).

The University of California at San Diego (San Diego, CA, USA) Institutional Review Board was responsible for the ethical oversight of the ABCD study. The secondary analysis of the data was approved by the Research Ethics Committee for the Royal College of Surgeons in Ireland.

### Participants

Of the 11,875 participants in the ABCD baseline sample, we selected only the 9,612 singleton-born individuals given systematic differences in birth weight and gestational age of multiple births. Presence of a twin (as reported by the primary caregiver in the Developmental History Questionnaire) OR presence of a co-twin/triplet within the ABCD study (rel_relationship) was used to define non-singleton. Siblings who did not share a womb were retained in the sample, with siblings statistically nested within the family unit (see Data Analysis). Mean age was 9.88 years (SD = 0.62). In 85% of cases, the primary respondent, henceforth referred to as "the parent”, was the biological mother of the child (10% biological fathers; 3% adoptive parents; 1% custodial parents; 1% other). The final sample size used in the analysis was 9,076 (M1) which was reduced in the fully-adjusted model (M3; 8,142).

### Measures

#### Birth Weight

Birth weight was reported by the parent in pounds and ounces, which was converted to kilograms. In some cases (N = 1622), pounds were reported whilst ounces were not— in these cases it was calculated to the nearest kilogram using pounds alone. Some cases (N = 683) reported in ounces but not in pounds—these values were very small and unlikely to be the true birth weight therefore were removed. One individual was removed from the analysis due to improbable birth weight for their gestational age (born 6 weeks early at 6.7 KG).

#### Gestational Age Groups

Children born with less than 28 weeks gestation were excluded from participating in the ABCD study. Further, parent-reported gestational age was only provided for children born earlier than 40 weeks, therefore we could not identify late-term or post-term births. We created 4 gestational age groups from the data available: full-term (39 + weeks; reference group), early-term (37–38 weeks), late preterm (34–36 weeks), and early-moderate preterm (33 weeks or less). Maternal retrospective recall of birth weight 9 years after the birth has been found to align closely with medical records (Rice et al., 2007). Descriptive statistics for these gestational age groups are provided in Table 1.

**Table 1:** Descriptive Statistics for all Categorical (left) & Continuous (right) Variables

***Categorical Variables***	*N*	% of Sample		***Continuous Variables***	Min–Max	Mean (SD)
Sex (Males:Females)	5,044:4,562	53:47%		Age (years)	9.0–10.9	9.9 (0.6)
GA group				Birth Weight (kgs)	0.91–6.41	3.35 (0.57)
Full-term (39 + wks)	8,585	91%		Maternal Age at Birth (years)	13–60	29 (6.32)
Early-term (37-38wks)	328	4%		Parental Education Level	1–7	4.5 (1.7)
Late preterm (34–36 wks)	408	4%		Parental Income Bracket	1–10	7.1 (2.5)
Early-moderate preterm (< = 33 wks)	148	2%		CBCL Total Problem Score	0–139	18.94 (18.32)
Single-Parent Family (Y:N)	1,888:7,611	20:80%		# Mental Health Issues in Family	0–8	2.3 (2.0)
Race/Ethnicity						
White	4,727	49%				
Hispanic	2,141	22%				
Black	1,465	15%				
Asian	242	3%				
Other	1,023	11%				

These groups were created in accordance with the literature and recommendations from leading bodies as follows. The World Health Organization (WHO) group “prematurity” into late-moderate preterm (32–37 weeks), very preterm (28–32 weeks) and extremely preterm (< 28 weeks) (Howson et al., 2013). Many have advocated the further subdivision of the late-moderate preterm group into a late preterm (34–36 weeks) and moderate preterm (32–33 weeks) (Engle et al., 2007; Raju et al., 2006) and the subdivision of term births into full-term (39–40 weeks) and early term (37–38 weeks) (Spong, 2013). Whilst group sizes for full-term, early-term and late preterm births were sufficient for analysis, group sizes for moderate preterm, very preterm and extremely preterm were very small (n = 98, 38, 26, respectively) therefore were combined into one early-moderate preterm group.

#### Child Behaviour Checklist (CBCL)

The CBCL (Achenbach & Rescorla, 2001) is a parent-rated questionnaire containing 119 items which are rated on a 3-point Likert scale (0 = not at all true; 1 = somewhat true; 2 = very true). Item scores were aggregated into 8 empirical sub-scales and a Total Problems score. The sub-scales are: (1) Anxious-Depressed, (2) Withdrawn-Depressed, (3) Somatic Complaints, (4) Social Problems, (5) Thought Problems, (6) Attention Problems, (7) Rule-Breaking Behavior & (8) Aggressive Behavior. We excluded the “Other Problems” scale given its non-specificity (items provided in Table S1). Note that the Attention Problems scale contains items pertaining to both inattention (e.g. “inattentive or easily distracted”) and hyperactivity (e.g. “can’t sit still, restless or hyperactive”). We added a 9th ASD (Autism Spectrum Disorder) sub-scale which includes 9 CBCL items, from the Social Problems (2 items), Thought Problems (3 items) and Withdrawn-Depressed (2 items), Anxious-Depressed (1 item) and Attention Problems domains (1 item; Table S1). This scale has demonstrated good discrimination between children with and without autism, even when the latter group includes children with ADHD and children collected from a clinical setting (Ooi et al., 2011; sensitivity 68–78%, specificity 73–92%). All CBCL scales were positively skewed (i.e. most individuals scored low) which is typical for typically-developing samples on this questionnaire (e.g. Kariuki et al., 2016; Polderman et al., 2007). The ABCD study provided CBCL total scores for participants who responded to *any* of the 112 items, regardless of missingness. We excluded 2 participants who did not have full data (one missing 4 items, the other missing majority of items).

#### Family History of Mental Illness

Parents were asked whether any blood relative of the child had ever experienced (1) depression, (2) problems with nerves, (3) mania, (4) psychosis, (5) drug abuse, (6) alcohol abuse (7) antisocial behavior, or whether a family member had (8) attempted/committed suicide. The specific wording of questions probing family history are provided in Supplementary Material. “Blood relatives” included biological parents & siblings, half-siblings, biological grandparents & aunts/uncles. We created summary variables such that, if there were 1 or more familial instances of an issue mentioned above, that type of issue was given a 1. That is, each mental health issue had a maximum score of 1. These were then summed to form a total score with a min–max of 0–8. This variable was thus a cumulative score of discrete mental health conditions in the extended family.

#### Socioeconomic Factors

Three socioeconomic factors were included in this study: household (combined parental) income, parental education level and single parenthood. Household income was captured by 10 annual income brackets from $5,000 or less to $200,000 or more. Parental educational level was captured by 7 levels from incomplete high school to doctoral degree. Dual-parenthood was qualified by presence of a partner who helped in raising the child and is involved in 40% or more of the child’s daily activities. Note this partner could be a spouse, boyfriend/girlfriend, relative or friend of the parent. Single-parenthood was the absence of such a partner. Greater detail on these variables is provided in Supplementary Material.

### Data Analysis

All analyses were performed in R (R Core Team, 2019) on version 3.6.2 ("Dark and Stormy Night”). A generalized linear mixed model with multivariate normal random effects was used for all analyses using Penalized Quasi-Likelihood (glmmPQL, MASS package). Nested random effects (subjects within families within sites) were required to capture non-independence of observations across siblings and data collection site (random =  ~ 1|site/family). CBCL scores (total problems and all 9 sub-scores) were highly positively skewed indicating we should not assume a normal distribution. We empirically demonstrated, using goodness-of-fit statistics (AIC, BIC), that the outcome data fit a gamma distribution better than a gaussian (Supplementary Information; Table S2). Changing the assumed distribution of the outcome but retaining an identity link meant all inputs linearly combined to estimate the outcome and that the outcome retained its original scaling (i.e. raw CBCL scale). A value of 1 was added to all CBCL scales to avoid 0’s as non-positive values are not allowed for the gamma family. Residual plots did not reveal any serious deviations from homoscedasticity.

This generalized linear mixed model (distribution: gamma, link: identity) was run with birth weight as a continuous predictor, CBCL score as continuous outcome and covariates adjusted for in consecutive models: M1 adjusted for sex only, M2 added race/ethnicity and socioeconomic factors (household income, parental education status, single parenthood), while M3 added family history of mental illness. All 3 models were re-run adjusting for gestational age (aim 3).

Specificity of the association between birth weight and mental health to attention problems (aim 1) was explored both visually, by plotting relative effect sizes of birth weight on all 9 CBCL sub-scores, and with inferential statistics by testing whether attention problems were significantly predicted by birth weight after correction for all covariates and multiple testing (Bonferroni corrected p = 0.05/9 = 0.006).

To test whether the effect of birth weight on mental health was moderated by sex (aim 2) we included a birth weight*sex interaction term to the fully-corrected model (M3) for the CBCL total problem score and all 9 sub-scores.

Given that some studies have found links between large birthweight and psychotic disorders (Brander et al., 2016; Keskinen et al., 2013; Lahti et al., 2015; Liuhanen et al., 2018) we explored whether the effect of birth weight on CBCL total problem score was better described as quadratic rather than linear by including a birth weight-squared term. We assessed the significance of the quadratic effect by observing the coefficient statistic and comparing model-fit statistics between equivalent linear and quadratic models (AIC, BIC).

## Results

Descriptive statistics in Table 1 show that 91% of children were born at full-term (39 weeks or more of gestation) and that the birth weight varied widely from sub-1 kg to over 6kgs with a mean of 3.35kgs. Mean CBCL total problem score was 18.94 out of a possible 238 and the max observed score was 139, suggesting mostly low but wide-ranging scores.

**Fig. 1:**
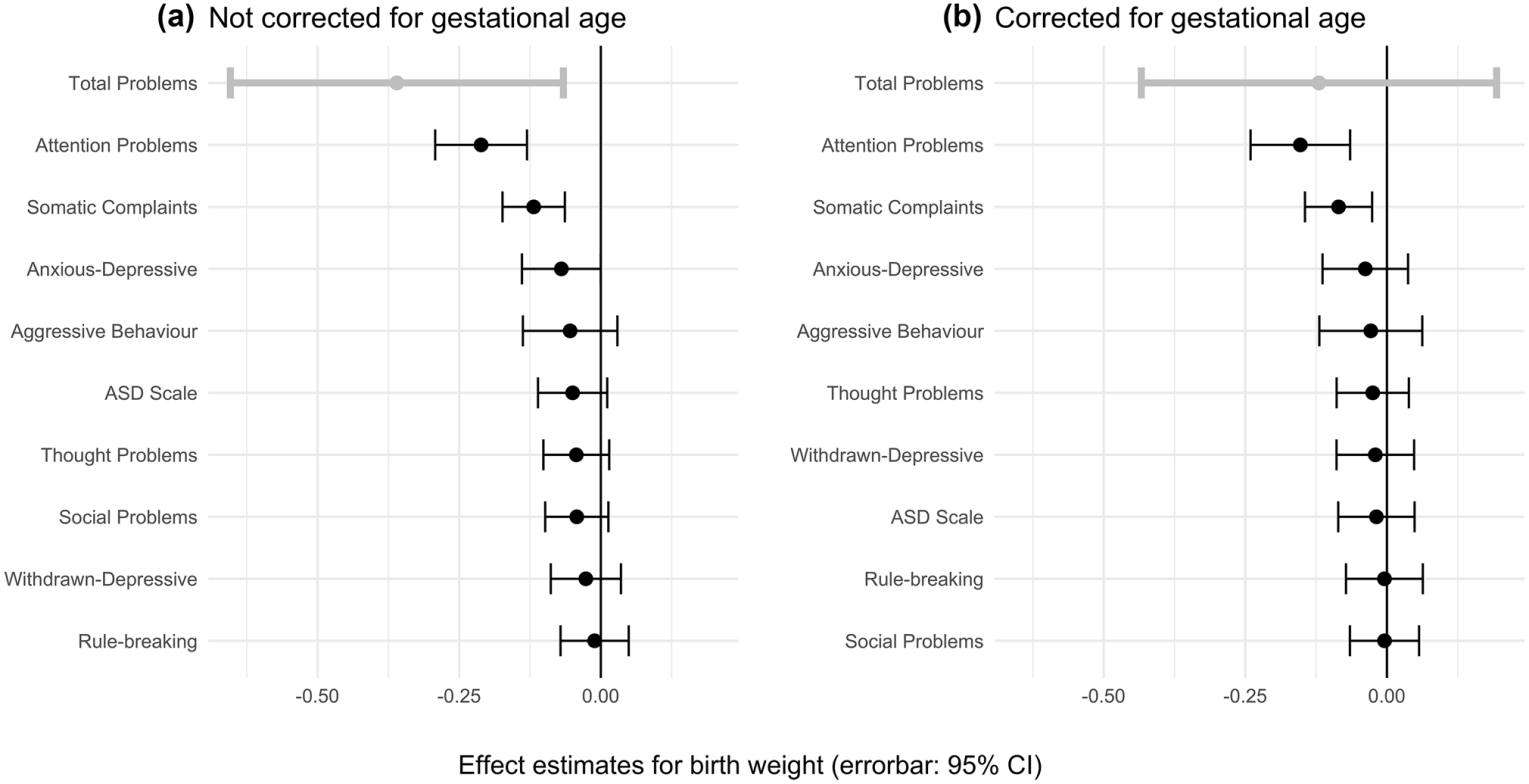
Effect of Birth Weight on each CBCL Scale, Before (**a**) & After (**b**) adjusting for Gestational Age. *Note:* The figure shows that only attention problems remains significantly associated with birth weight after adjusting for gestational age. Sub-scales are listed in order of effect (standardized beta). All estimates taken from fully adjusted model (M3)

Figure 1 shows that, attention problems was the CBCL sub-score with the strongest linear association with birth weight. All estimates shown in this figure were controlled for socioeconomic factors, race/ethnicity and familial mental health history. Figure 1A also shows that before controlling for gestational age there were significant effects of birth weight on total problems (β = -0.36, SE = 0.15, p = 0.02) but that this become non-significant after controlling for gestational age (Fig. 1B). Attention problems and somatic complaints were the only measured outcomes to remain significantly associated with birth weight after controlling for gestational age and all potential confounders (attention: β = -0.15, SE = 0.05, p = 0.001; somatic: β = -0.09, SE = 0.03, p = 0.005). These estimates also met the Bonferroni-corrected threshold for significance (p = 0.006). There were no significant associations between birth weight and the ASD scale (Table S4).

Table 2 shows how each level of adjustment for potential confounds (M1-M3) and adjustment for gestational age affected the linear association between birth weight and the CBCL total problem scale. It shows that covarying for gestational age resulted in a large drop in the effect size of birth weight on the total problem score suggesting that it was mainly gestational-age-linked variation in birth weight that drove this effect (also see Fig. 1). Table 2 also shows an unexpected non-linear effect of gestational age on total problems such that those born at term but slightly early (early-term group; 37–38 weeks) had the highest problem scores relative to those born full-term (39 + weeks; also see Fig. S1). A series of sensitivity analyses showed this group effect on CBCL total problems to be robust (Fig S2-S4). Effect estimates for all other covariates (e.g. family income) on CBCL total problems are provided in Table S3. There were no significant quadratic effects of birth weight on the CBCL total problems scale or on most sub-scales. However the quadratic relationship between birth weight and aggressive behavior was significant (Table S5), following an inverted-U shape whereby average birth weight was linked with higher aggressive behaviour (Fig. S2).

**Table 2:** Effect of Birth Weight and Gestational Age on CBCL Total Problems. Shown are Beta Estimates [and 95% Confidence Intervals], t-statistics and p-values from Wald Tests

	**M1**	**M2**	**M3**
***N***	**9,076**	**8,208**	**8,142﻿**
	Adjusted for **sex**	M1 + Adjusted for socioeconomic factors & race/ethnicity	M2 + Adjusted for family history of mental illness
Birth weight	-0.43 [-0.74, -0.12]**	-0.69 [-1.04, -0.34]***	-0.36 [-0.65, -0.06]*
	*t* = -2.70, *p* = 0.007	*t* = -3.90, *p* < 0.001	*t* = -2.35, *p* = 0.02
Birth weight (controlling for gestational age group)	0.02 [-0.32, 0.36]	-0.11 [-0.49, 0.26]	-0.12 [-0.44, 0.21]
	*t* = 0.12, *p* = 0.91	*t* = -0.58, *p* = 0.56	*t* = -0.71, *p* = 0.48
Gestational age group^a^ (controlling for birth weight)			
Early-term (37–38 wks)	6.23 [4.84, 7.63]***	4.82 [3.44, 6.20]***	3.34 [2.10, 4.57]***
	*t* = 8.76, *p* < 0.001	*t* = 6.88, *p* < 0.001	*t* = 5.30, *p* < 0.001
Late preterm (34–36 wks)	3.66 [2.50, 4.81]***	3.73 [2.47, 4.98]***	1.83 [0.85, 2.82]***
	*t* = 6.22, *p* < 0.001	*t* = 5.83, *p* < 0.001	*t* = 3.64, *p* < 0.001
Early-moderate preterm (≤ 33 wks)	1.11 [-0.54, 2.76]	2.21 [0.22, 4.19]*	0.43 [-1.21, 2.06]
	*t* = 1.32, *p* = 0.19	*t* = 2.18, *p* = 0.03	*t* = 0.51, *p* = 0.61

Beta estimates are in original units of the CBCL. M1-M3 refer to models with increasing numbers of covariates

^*^p < .05; **p < .01; ***p < .001

^a^Reference group: Full-term (39 + weeks gestation)

**Fig. 2:**
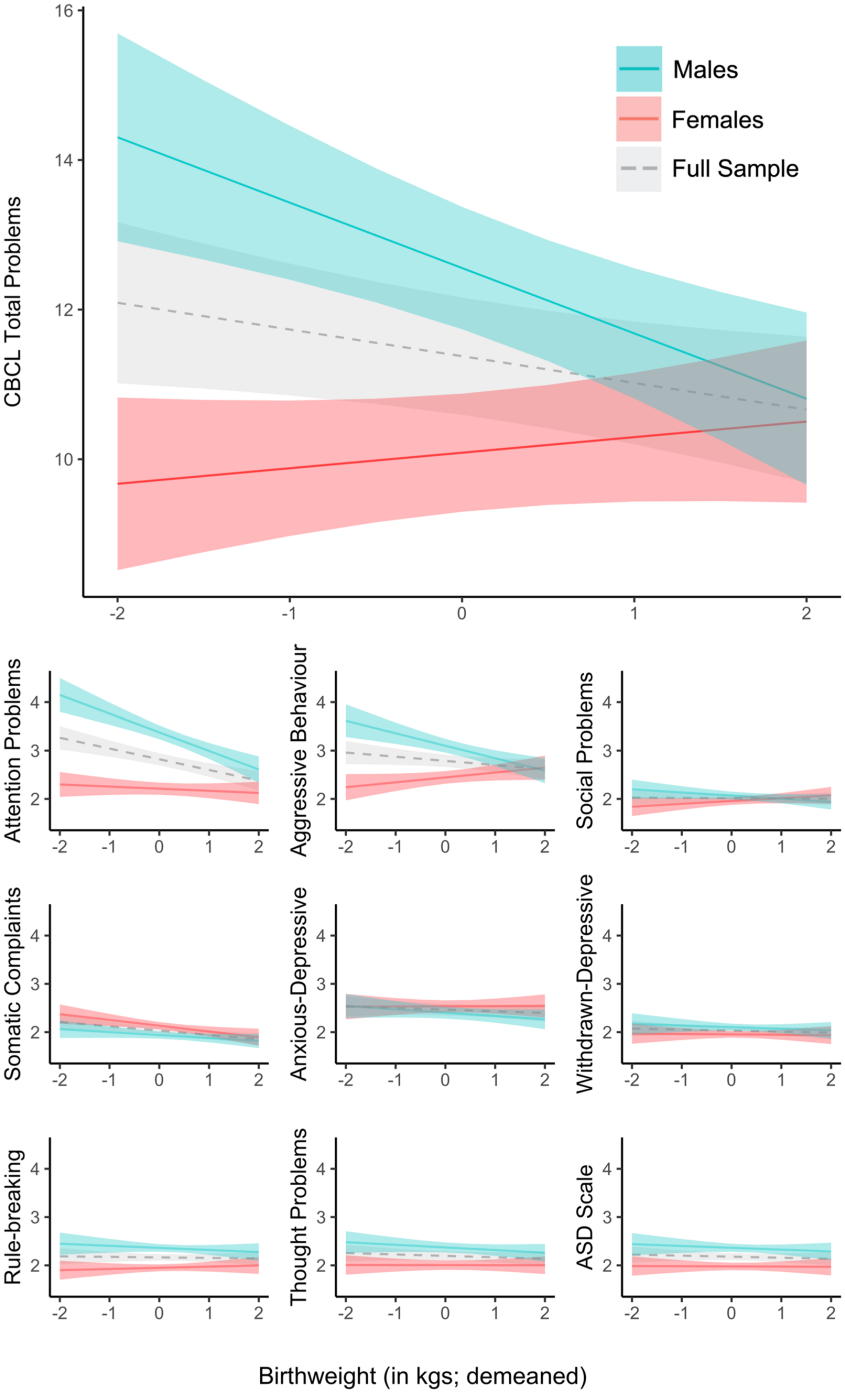
Interactions Between Birth Weight and Sex on CBCL Total Problems Scale and all 9 Sub-Scales. *Note:* Significant interaction found between birth weight and sex on total problems and 2 sub-scales (attention problems and aggressive behavior). Males drove the negative association between birth weight and these outcomes. Plots show effect estimates from fully-adjusted model (M3), including adjustment for gestational age. Error bands reflect 95% confidence interval

Sex moderated the effect of birth weight on total problems such that the inverse association observed in the full sample was driven by males (Fig. 2; fully adjusted interaction effect β = -1.24, SE = 0.31, t = -4.05, p < 0.001). Covarying for gestational age did not change this interaction effect drastically (β = -1.08, SE = 0.31, t = -3.51, p < 0.001). With regard to sub-scales, the interaction between sex and birth weight was significant only for attention problems (β = -0.35, SE = 0.08, t = -4.16, p < 0.001) and aggressive behavior (β = -0.38, SE = 0.08, t = -4.47, p < 0.001), both of which survived Bonferroni correction. There was a weaker non-significant interaction of sex and birth weight on social problems (β = -0.13, SE = 0.06, t = -2.31, p = 0.02) which was not significant at the corrected level (p = 0.006). Adjusting for gestational age did not drastically change the interactive effect estimates of sex and birth weight on attention problems (β = -0.34, SE = 0.09, t = -4.00, p < 0.001), aggressive behavior (β = -0.36, SE = 0.08, t = -4.25, p < 0.001) or social problems (β = -0.12, SE = 0.06, t = -2.22, p = 0.03). There was no significant interaction between sex and birth weight on the ASD scale (Fig. 2).

## Discussion

### Specificity to Attention Problems

Comparing relative effects of birth weight on 9 CBCL subscales, birth weight had the strongest effect on the attention problem scale in 9–10 year old children (Fig. 1A), supporting the specificity of this association. This association survived adjustment for gestational age, race/ethnicity, socioeconomics (family income, parental education, single parenthood), and family history of mental illness (β = -0.15, SE = 0.05, t = -3.41, p < 0.001; Fig. 1B). We also found that birth weight was significantly correlated with the somatic complaints subscale, which survived adjustment for gestational age and potential confounds (β = -0.09, SE = 0.03, t = -2.82, p = 0.005). The items of the somatic complaints scale ask how often the child experiences headaches, tiredness, nausea, stomach aches, etc. and may therefore be better described as a measure of physical (rather than psychological) wellbeing. This interpretation is supported by the associations between low birth weight and markers of physical health such as inflammation and cardiometabolic risk (Belbasis et al., 2016; Tzoulaki et al., 2008).

Unlike two recent Swedish population registry-based studies, which found the strongest relative effect of birth weight on autism (Class et al., 2014; Pettersson et al., 2019), we did not find birth weight was significantly correlated with a CBCL-based ASD scale or the social problem scale even at an uncorrected p-threshold. Several differences between our study and those may explain differences in results including our younger sample, a narrower age-range (e.g. Pettersson et al. (2019) mean age = 27, range = 14–40) and our use of questionnaire-based scales rather than diagnostic data. Registry studies are limited to psychiatric outcomes listed in national hospital records and outcomes are thus dichotomous, do not account for comorbidities and only include those who are unwell enough to be hospitalized. It’s therefore also possible the reported associations between birth weight and autism (Class et al., 2014; Pettersson et al., 2019) were confounded by concurrent or sequential comorbidity with ADHD (Gargaro et al., 2011; Leyfer et al., 2006; Rao & Landa, 2014). The discordance between our findings and two large psychiatric registry-based studies highlights the importance of assimilating evidence from different study designs.

Other studies have identified associations between birth weight and non-neurodevelopmental outcomes such as psychotic/bipolar disorder and depression (Class et al., 2014; De Mola et al., 2014; Lahti et al., 2015; Larsen et al., 2010; Pettersson et al., 2019). Sample age may also explain why we did not find an association between birth weight and withdrawn-depressed, anxious-depressed or thought problem scales. Age in our study ranged narrowly from 9 to 10 years old (mean age = 9.9) and the mean age of samples in the aforementioned studies are generally in early-mid adulthood, with wider ranges. Severe affective and psychotic disorders would likely be rare in children as young as 9–10. Birth weight-linked attention problems in childhood may increase the risk of other psychopathology over time though this trajectory has not been tested to our knowledge.

### Sex Differences

The effects of birth weight on CBCL total problems, attention problems and aggressive behavior were driven by males (Fig. 2). The sex-dependency of the birth weight effect on mental health is not well established in the literature. Just one study, also in U.S. children, found that sex moderated the effect of birth weight on symptoms of ADHD (Momany et al., 2017). They found inattention symptoms showed a sex-by-birth-weight interaction, with a stronger inverse association in males. While the CBCL attention problem scale used in our study contains a mix of inattention and hyperactivity items, the interaction effect on this scale (*β* = 0.35, 95% CI = 0.19–0.51) is very similar that of Momany et al.’s on parent- and teacher-rated inattentive symptoms (*β* = 0.34, 95% CI = 0.13–0.55) both in terms of magnitude and variance. We extended this pattern to two more outcomes: aggressive behavior and general mental health (CBCL total problems; Fig. 2). We also found that the male-driven effect of birth weight on these problems was not explained by gestational age, suggesting that males are at greater risk to restricted foetal growth specifically.

One plausible biological basis for this sex difference is that the limbic system, critical for emotional regulation (among other things), is more susceptible to prenatal insults (e.g. ischemia-hypoxia) in males compared to females. Rat experiments have shown that withdrawal of oxygen supply perinatally impairs behaviour and alters prefrontal dopamine (and other monoamine) levels in males more than females (Laplante et al., 2012; Amanda L. Smith et al., 2014). A human twin study found that birth weight discrepancy was linked with functional connectivity in the limbic network (lower birth weight—less efficient connectivity), an effect that was stronger in males compared to females (Hayward et al., 2020). Sex differences in microglial activation and inflammatory responses may underlie this male vulnerability to prenatal hypoxic-ischemic events (Mirza et al., 2015).

Our findings, and those of Momany et al. (2017), may be cohort-specific or United States-specific as studies in other populations have not found birth weight to have stronger effects on attention problems in males compared to females. In a Swedish register-based study (546,894 sibling pairs), male-male sibling pairs had similar estimates as female-female sibling pairs for the effect of birth weight on neurodevelopmental problems and general psychopathology (Pettersson et al., 2019) and in a Brazilian birth cohort (N = 3749), being low birth weight and small-for-gestational age was associated with CBCL attention problems in 4-year-old girls, but not in boys (Murray et al., 2015).

The sex-dependent effect of birth weight on general mental health and disruptive problems has potentially important implications for our understanding and treatment of childhood psychopathology, even if limited to certain populations. These findings should be replicated in both human observational and animal experimental studies before generalization.

### Gestational Age vs Foetal Growth

The strength of the association between birth weight and attention problems attenuated slightly but remained significantafter adjustment for gestational age (before adjustment: β = -0.21, SE = 0.04, p < 0.001; after adjustment: β = -0.15, SE = 0.04, p < 0.001) implying this effect was driven largely by foetal growth, consistent with other findings (Ficks et al., 2013; Groen-Blokhuis et al., 2011; Hultman et al., 2007; Momany et al., 2018; Pettersson et al., 2015). Though in boys, the effect of birth weight on total problems remained significant even after adjustment for gestational age (Fig. 2).

We observed an atypical non-linear effect of gestational age on CBCL total problem and some sub-scores. The early-term group (born at 37–38 weeks) had the worst CBCL outcomes (Table 2; Fig. S1). This was unexpected and is in contrast to many studies which have found the risk of mental illness to increase linearly with the extent of prematurity (Bhutta et al., 2002; Lindström et al., 2009; Nosarti et al., 2012). Figure S1 shows that the early term group were characterized by particularly high scores in the Anxious-Depressive domain. We explored some potential explanations for this result in sensitivity analyses and showed that this non-linear effect was not explained by unequal gestational age group sizes (Fig. S3), disproportionate number of males (Table S6; Fig. S4) or by the presence of CBCL outliers in the early-term group (Fig. S5). One possible explanation for this result is the context in which participants were born. This period in the U.S. (2006–08) is characterized by high rates of marginally-indicated or elective inductions in early-term births: the rates of induced labour in the U.S. more than doubled between 1991 and 2006 from ~ 10% to ~ 22% (Martin et al., 2010) which likely contributed to the increased prevalence of late preterm births at this time (American College of Obstetricians & Gynecologists, 2009; MacDorman et al., 2010). This may be relevant to our findings as induced vaginal deliveries have been linked with behavioral problems at age 7, though this finding did not survive correction for all covariates (Curran et al., 2016). Another study found induced, or augmented, labor to increase the odds of autism (Gregory et al., 2013) though there have been failures to replicate this after controlling for familial factors (Oberg et al., 2016). And oxytocin, which is administered intravenously to induce or augment labor, has shown a small but significant dose–response association with CBCL total problems across childhood (adjusted odds ratio = 1.03 [95% CI: 1.01–1.06]; (Guastella et al., 2018). In conclusion, there is (a) mixed evidence as to whether induced labor influences the mental health of children and (b) insufficient evidence in the ABCD study to explore whether this term-born group (37–38 weeks) had higher rates of induced labor or whether this accounts for their higher CBCL total problem scores. Future data collection should retrospectively collect mode of delivery data from ABCD parents and the association between slightly early term birth and poor mental health should be explored in other U.S. based cohorts.

## Strengths & Limitations

The strengths of this study include the large sample of children (N > 8,000) within a narrow age range (9–10 years), the continuous rather than binary measurement of birth weight and mental health allowing us to capture smaller effects on a continuous scale, and the use of generalized linear model to predict non-normally distributed mental health scales. Limitations of this study include the non-generalizability of findings to multiple births and the reliance on parent-report only for both retrospective birth data and the child’s mental health status. The ASD scale constructed from CBCL items (Ooi et al., 2011) also had limitations, specifically its overlap with other scales (e.g. social and thought problems) and the absence of several key signs and symptoms of autism (e.g. restricted interests, sensory hypersensitivity, recognition of emotions). Future studies should use multiple informants (e.g. parent, child, teacher) to form a more unbiased snapshot of the child’s mental health and should validate parent-report birth data against birth records. The null association between birth weight and the CBCL ASD scale should also be replicated using an alternative measure of autistic traits (e.g. Autism Spectrum Quotient; (Auyeung et al., 2008)).

## Conclusion

Our results suggest that the psychological effects of birth weight are strongest for attention problems at this age (9–10 years), rather than autistic traits. This contrasts with recent registry-based findings and highlights the importance of assimilating evidence from a variety of study designs to avoid sampling bias. Our data also suggests males are particularly vulnerable to the psychological effects of lower birth weight, particularly problems of attention and aggression. This, given further replication, may have strong implications for sex-specific mechanistic and prediction models.

## Supplementary Information

Below is the link to the electronic supplementary material.

Supplementary file1 (DOCX 61435 KB)

## Data Availability

Data used in this study is from the Adolescent Brain Cognitive Development (ABCD) Study which is stored in the NIMH Data Archive (NDA) Repository (ndar.nih.gov). The ABCD data repository grows and changes over time: the data used in this report was drawn from Release 3.0 (https://doi.org/10.15154/1519007) and will be available on the NDA as a link to this study (https://doi.org/10.15154/1520466).

## Code Availability

The MATLAB and R code used to clean the data and perform analysis will be provided, along with the registered study, on the NDA repository (https://doi.org/10.15154/1520466).

## References

[CR1] Aarnoudse-Moens CSH, Weisglas-Kuperus N, van Goudoever JB, Oosterlaan J (2009). Meta-analysis of neurobehavioral outcomes in very preterm and/or very low birth weight children. Pediatrics.

[CR2] Abel KM, Wicks S, Susser ES, Dalman C, Pedersen MG, Mortensen PB (2010). Birth weight, schizophrenia, and adult mental disorder: Is risk confined to the smallest babies?. Archives of General Psychiatry.

[CR3] Achenbach T, Rescorla L (2001). Manual for the ASEBA school-age forms & profiles: An integrated system of multi-informant assessment Burlington.

[CR4] American College of Obstetricians and Gynecologists (2009). Practice Bulletin No. 107: Induction of Labor. Obstetrics and Gynecology.

[CR5] Auyeung B, Baron-Cohen S, Wheelwright S, Allison C (2008). The autism spectrum quotient: Children’s version (AQ-Child). Journal of Autism and Developmental Disorders.

[CR6] Belbasis L, Savvidou MD, Kanu C, Evangelou E, Tzoulaki I (2016). Birth weight in relation to health and disease in later life: An umbrella review of systematic reviews and meta-analyses. BMC Medicine.

[CR7] Bhutta AT, Cleves MA, Casey PH, Cradock MM, Anand K (2002). Cognitive and behavioral outcomes of school-aged children who were born preterm: A meta-analysis. Journal of the American Medical Association.

[CR8] Brander G, Rydell M, Kuja-Halkola R, de la Cruz LF, Lichtenstein P, Serlachius E (2016). Association of perinatal risk factors with obsessive-compulsive disorder: A population-based birth cohort, sibling control study. Journal of the American Medical Association: Psychiatry.

[CR9] Burnett A, Anderson P, Cheong J, Doyle L, Davey C, Wood S (2011). Prevalence of psychiatric diagnoses in preterm and full-term children, adolescents and young adults: A meta-analysis. Psychological Medicine.

[CR10] Caspi A, Moffitt TE (2018). All for One and One for All: Mental Disorders in One Dimension. American Journal of Psychiatry.

[CR11] Class QA, Rickert ME, Larsson H, Lichtenstein P, D'Onofrio BM (2014). Fetal growth and psychiatric and socioeconomic problems: Population-based sibling comparison. The British Journal of Psychiatry.

[CR12] Cross-Disorder Group of the Psychiatric Genomics Consortium. (2013). Identification of risk loci with shared effects on five major psychiatric disorders: A genome-wide analysis. *The Lancet,**381*(9875), 1371–1379. https://www.sciencedirect.com/science/article/pii/S0140673612621291#! 10.1016/S0140-6736(12)62129-1 PMC3714010 23453885

[CR13] Curran EA, Cryan JF, Kenny LC, Dinan TG, Kearney PM, Khashan AS (2016). Obstetrical Mode of Delivery and Childhood Behavior and Psychological Development in a British Cohort. Journal of Autism and Developmental Disorders.

[CR14] Davies C, Segre G, Estradé A, Radua J, De Micheli A, Provenzani U (2020). Prenatal and perinatal risk and protective factors for psychosis: A systematic review and meta-analysis. The Lancet Psychiatry.

[CR15] De Mola CL, De França GVA, de Avila Quevedo L, Horta BL (2014). Low birth weight, preterm birth and small for gestational age association with adult depression: Systematic review and meta-analysis. The British Journal of Psychiatry.

[CR16] DiPietro JA, Voegtline KM (2017). The gestational foundation of sex differences in development and vulnerability. Neuroscience.

[CR17] Engle WA, Tomashek KM, Wallman C, Committee on, F., & Newborn, A. A. o. P.  (2007). "Late-preterm" infants: A population at risk. Pediatrics.

[CR18] Ficks CA, Lahey BB, Waldman ID (2013). Does low birth weight share common genetic or environmental risk with childhood disruptive disorders?. Journal of Abnormal Psychology.

[CR19] Garavan H, Bartsch H, Conway K, Decastro A, Goldstein R, Heeringa S (2018). Recruiting the ABCD sample: Design considerations and procedures. Developmental Cognitive Neuroscience.

[CR20] Gargaro BA, Rinehart NJ, Bradshaw JL, Tonge BJ, Sheppard DM (2011). Autism and ADHD: How far have we come in the comorbidity debate?. Neuroscience and Biobehavioral Reviews.

[CR21] Gregory SG, Anthopolos R, Osgood CE, Grotegut CA, Miranda ML (2013). Association of Autism With Induced or Augmented Childbirth in North Carolina Birth Record (1990–1998) and Education Research (1997–2007) Databases. JAMA Pediatrics.

[CR22] Groen-Blokhuis MM, Middeldorp CM, van Beijsterveldt CE, Boomsma DI (2011). Evidence for a causal association of low birth weight and attention problems. Journal of the American Academy of Child and Adolescent Psychiatry.

[CR23] Guastella AJ, Cooper MN, White CRH, White MK, Pennell CE, Whitehouse AJO (2018). Does perinatal exposure to exogenous oxytocin influence child behavioural problems and autistic-like behaviours to 20 years of age?. Journal of Child Psychology and Psychiatry.

[CR24] Hayward DA, Pomares F, Casey KF, Ismaylova E, Levesque M, Greenlaw K (2020). Birth weight is associated with adolescent brain development: A multimodal imaging study in monozygotic twins. Human Brain Mapping.

[CR25] Heaman M, Kingston D, Chalmers B, Sauve R, Lee L, Young D (2013). Risk Factors for Preterm Birth and Small-for-gestational-age Births among C anadian Women. Paediatric and Perinatal Epidemiology.

[CR26] Hill AB (1965). The Environment and Disease: Association or Causation?. Proceedings of the Royal Society of Medicine.

[CR27] Howson CP, Kinney MV, McDougall L, Lawn JE (2013). Born too soon: Preterm birth matters. Reproductive Health.

[CR28] Hultman CM, Torrang A, Tuvblad C, Cnattingius S, Larsson JO, Lichtenstein P (2007). Birth weight and attention-deficit/hyperactivity symptoms in childhood and early adolescence: A prospective Swedish twin study. Journal of the American Academy of Child and Adolescent Psychiatry.

[CR29] Jensen CM, Steinhausen H-C (2015). Comorbid mental disorders in children and adolescents with attention-deficit/hyperactivity disorder in a large nationwide study. Attention Deficit and Hyperactivity Disorders.

[CR30] Kariuki SM, Abubakar A, Murray E, Stein A, Newton CRJC (2016). Evaluation of psychometric properties and factorial structure of the pre-school child behaviour checklist at the Kenyan Coast. Child and Adolescent Psychiatry and Mental Health.

[CR31] Keskinen E, Miettunen J, Koivumaa-Honkanen H, Mäki P, Isohanni M, Jääskeläinen E (2013). Interaction between parental psychosis and risk factors during pregnancy and birth for schizophrenia—the Northern Finland 1966 Birth Cohort study. Schizophrenia Research.

[CR32] Lahti M, Eriksson J, Heinonen K, Kajantie E, Lahti J, Wahlbeck K (2015). Late preterm birth, post-term birth, and abnormal fetal growth as risk factors for severe mental disorders from early to late adulthood. Psychological Medicine.

[CR33] Lang, J. M., Lieberman, E., & Cohen, A. (1996). A comparison of risk factors for preterm labor and term small-for-gestational-age birth. *Epidemiology*, 369–376. 10.1097/00001648-199607000-00006 8793362

[CR34] Laplante F, Brake WG, Chehab SL, Sullivan RM (2012). Sex differences in the effects of perinatal anoxia on dopamine function in rats. Neuroscience Letters.

[CR35] Larsen JK, Bendsen BB, Foldager L, Munk-Jørgensen P (2010). Prematurity and low birth weight as risk factors for the development of affective disorder, especially depression and schizophrenia: A register study. Acta Neuropsychiatrica.

[CR36] Leyfer OT, Folstein SE, Bacalman S, Davis NO, Dinh E, Morgan J (2006). Comorbid psychiatric disorders in children with autism: Interview development and rates of disorders. Journal of Autism and Developmental Disorders.

[CR37] Lindström K, Lindblad F, Hjern A (2009). Psychiatric morbidity in adolescents and young adults born preterm: A Swedish national cohort study. Pediatrics.

[CR38] Liuhanen J, Suvisaari J, Kajantie E, Miettunen J, Sarin A-P, Järvelin M-R (2018). Interaction between compound genetic risk for schizophrenia and high birth weight contributes to social anhedonia and schizophrenia in women. Psychiatry Research.

[CR39] MacDorman MF, Declercq E, Zhang J (2010). Obstetrical intervention and the singleton preterm birth rate in the United States from 1991–2006. American Journal of Public Health.

[CR40] Martin, J. A., Hamilton, B. E., Sutton, P. D., Ventura, S. J., Mathews, T., Kirmeyer, S., et al. (2010). Births: final data for 2007. 21254725

[CR41] Mathewson KJ, Chow CH, Dobson KG, Pope EI, Schmidt LA, Van Lieshout RJ (2017). Mental health of extremely low birth weight survivors: A systematic review and meta-analysis. Psychological Bulletin.

[CR42] Mirza MA, Ritzel R, Xu Y, McCullough LD, Liu F (2015). Sexually dimorphic outcomes and inflammatory responses in hypoxic-ischemic encephalopathy. Journal of Neuroinflammation.

[CR43] Momany AM, Kamradt JM, Nikolas MA (2018). A Meta-Analysis of the Association Between Birth Weight and Attention Deficit Hyperactivity Disorder. Journal of Abnormal Child Psychology.

[CR44] Momany AM, Kamradt JM, Ullsperger JM, Elmore AL, Nigg JT, Nikolas MA (2017). Sex moderates the impact of birth weight on child externalizing psychopathology. Journal of Abnormal Psychology.

[CR45] Murray E, Matijasevich A, Santos IS, Barros AJ, Anselmi L, Barros FC (2015). Sex differences in the association between foetal growth and child attention at age four: Specific vulnerability of girls. Journal of Child Psychology and Psychiatry.

[CR46] Nosarti C, Reichenberg A, Murray RM, Cnattingius S, Lambe MP, Yin L (2012). Preterm birth and psychiatric disorders in young adult life. Archives of General Psychiatry.

[CR47] Oberg AS, D’Onofrio BM, Rickert ME, Hernandez-Diaz S, Ecker JL, Almqvist C (2016). Association of Labor Induction With Offspring Risk of Autism Spectrum Disorders. JAMA Pediatrics.

[CR48] Ooi YP, Rescorla L, Ang RP, Woo B, Fung DS (2011). Identification of autism spectrum disorders using the Child Behavior Checklist in Singapore. Journal of Autism and Developmental Disorders.

[CR49] Pettersson, E., Larsson, H., D'Onofrio, B., Almqvist, C., & Lichtenstein, P. (2019). Association of Fetal Growth With General and Specific Mental Health Conditions. *Journal of the American Medical Association: Psychiatry*. 10.1001/jamapsychiatry.2018.4342 10.1001/jamapsychiatry.2018.4342 PMC6495458 30725083

[CR50] Pettersson E, Sjölander A, Almqvist C, Anckarsäter H, D'Onofrio BM, Lichtenstein P (2015). Birth weight as an independent predictor of ADHD symptoms: A within-twin pair analysis. Journal of Child Psychology and Psychiatry.

[CR51] Polderman TJ, Derks EM, Hudziak JJ, Verhulst FC, Posthuma D, Boomsma DI (2007). Across the continuum of attention skills: A twin study of the SWAN ADHD rating scale. Journal of Child Psychology and Psychiatry.

[CR52] R Core Team (2019). *R: A language and environment for statistical computing*. R Foundation for Statistical Computing, Vienna, Austria. https://www.R-project.org/

[CR53] Raju TN, Higgins RD, Stark AR, Leveno KJ (2006). Optimizing care and outcome for late-preterm (near-term) infants: A summary of the workshop sponsored by the National Institute of Child Health and Human Development. Pediatrics.

[CR54] Rao PA, Landa RJ (2014). Association between severity of behavioral phenotype and comorbid attention deficit hyperactivity disorder symptoms in children with autism spectrum disorders. Autism.

[CR55] Rice F, Lewis A, Harold G, van den Bree M, Boivin J, Hay DF (2007). Agreement between maternal report and antenatal records for a range of pre and peri-natal factors: The influence of maternal and child characteristics. Early Human Development.

[CR56] Selzam S, Coleman JRI, Caspi A, Moffitt TE, Plomin R (2018). A polygenic p factor for major psychiatric disorders. Translational Psychiatry.

[CR57] Smith AL, Alexander M, Rosenkrantz TS, Sadek ML, Fitch RH (2014). Sex differences in behavioral outcome following neonatal hypoxia ischemia: Insights from a clinical meta-analysis and a rodent model of induced hypoxic ischemic brain injury. Experimental Neurology.

[CR58] Smith TF, Schmidt-Kastner R, McGeary JE, Kaczorowski JA, Knopik VS (2016). Pre- and Perinatal Ischemia-Hypoxia, the Ischemia-Hypoxia Response Pathway, and ADHD Risk. Behavior Genetics.

[CR59] Spong CY (2013). Defining “term” pregnancy: Recommendations from the Defining “Term” Pregnancy Workgroup. Journal of the American Medical Association.

[CR60] Tzoulaki I, Jarvelin M-R, Hartikainen A-L, Leinonen M, Pouta A, Paldanius M (2008). Size at birth, weight gain over the life course, and low-grade inflammation in young adulthood: Northern Finland 1966 Birth Cohort study. European Heart Journal.

[CR61] Vu HD, Dickinson C, Kandasamy Y (2018). Sex difference in mortality for premature and low birth weight neonates: A systematic review. American Journal of Perinatology.

[CR62] Wiles NJ, Peters TJ, Heron J, Gunnell D, Emond A, Lewis G (2006). Fetal growth and childhood behavioral problems: Results from the ALSPAC cohort. American Journal of Epidemiology.

[CR63] Yoshimasu K, Barbaresi WJ, Colligan RC, Voigt RG, Killian JM, Weaver AL (2012). Childhood ADHD is strongly associated with a broad range of psychiatric disorders during adolescence: A population-based birth cohort study. Journal of Child Psychology and Psychiatry.

