# Supplementary material for: Birth Weight and Childhood Psychopathology in the ABCD Cohort: Association is Strongest for Attention Problems and is Moderated by Sex
Source: Res Child Adolesc Psychopathol. 2022 Jan 24;50(5):563–75. doi: 10.1007/s10802-021-00859-0 (PMC9054906; doi:10.1007/s10802-021-00859-0)
Supplement: Supplementary file 1 — Supplementary file1 (DOCX 61435 KB) [file 10802_2021_859_MOESM1_ESM.docx]

Supplementary Material

**Table S1.** CBCL items and their corresponding scales.

| Scale | Items |
| --- | --- |
| Anxious-Depressed | 14. Cries a lot; 29. Fears; 30. Fears going to school; 31. Fears might do something do bad; 32. Feels he/she has to be perfect; 33. Feels unloved; 35. Feels worthless/inferior; 45. Nervous, high-strung, tense; 50. Too fearful/anxious; 52. Feels too guilty; 71. Self-conscious/easily embarrassed; 91. Thinks about killing self; 112. Worries |
| Withdrawn-Depressed | 5. Enjoys little; 42. Rather be alone; 65. Won’t talk; 69. Secretive; 75. Too shy/timid; 102. Lacks Energy; 103. Sad; 111. Withdrawn |
| Somatic Complaints | 47. Nightmares; 49. Constipated; 51. Dizzy; 54. Tired; 56a. Aches; 56b. Headaches; 56c. Nausea; 56d. Eye problems; 56e. Skin problems; 56f. Stomach aches; 56g. Vomits |
| Social Problems | 11. Dependent; 12. Lonely; 25. Doesn’t get along with other kids; 27. Jealous; 34. Feels others are out to get him/her; 36. Accidents; 38. Teased; 48. Not liked; 62. Clumsy; 64. Prefers being with younger kids; 79. Speech problems |
| Attention Problems | 1. Acts too young; 4. Fails to finish things he/she started; 8. Can’t concentrate; 10. Can’t sit still; 13. Confused; 17. Daydreams; 41. Impulsive; 61. Poor school work; 78. Inattentive; 80. Stares blankly |
| Thought Problems | 9. Obsessions; 18. Harms self; 40. Hears things; 46. Nervous movements/twitching; 58. Picks skin; 59. Plays with own sex parts in public; 60. Plays with own sex parts too much; 66. Repeats acts over and over; 70. Sees things that aren’t there; 76. Sleeps less than most kids; 83. Stores things he/she doesn’t need; 84. Strange behavior; 85. Strange ideas; 92. Sleep walk; 100. Sleep problems |
| Rule-Breaking Behavior | 2. Alcohol; 26. Lacks guilt; 28. Breaks rules; 39. Bad friends; 43. Lies cheats; 63. Prefer older; 67. Run away; 72. Sets fires; 73. Sexual problems; 81. Steals at home; 82. Steals outside home; 90. Swears; 96. Thinks about sex too much; 99. Tobacco; 101. Truant; 105. Uses drugs; 106. Vandalism |
| Aggressive Behavior | 3. Argues; 16. Mean; 19. Demands attention; 20. Destroy own; 21. Destroy other; 22. Disobey home; 23. Disobey school; 37. Fights; 57. Attacks; 68. Screams; 86. Stubborn; 87. Mood changes; 88. Sulks; 89. Suspicious; 94. Teases; 95. Temper; 97. Threaten; 104. Loud |
| ASD scale* | 1. Acts too young; 25. Doesn’t get along with other kids; 29. Fears; 42. Rather be alone; 46. Nervous movements/twitching; 66. Repeats acts over and over; 79. Speech problem; 84. Strange behavior; 111. Withdrawn |
| *Autism spectrum scale is not one of the original empirically-validated CBCL syndrome scales (Achenbach and Rescorla, 2001) but was validated by Ooi et al., 2011. | |

## Details of Socioeconomic Factors

### Household Income

Parents of participating children were asked the following question: “What is your total combined family income for the past 12 months? This should include income (before taxes and deductions) from all sources, wages, rent from properties, social security, disability and/or veteran's benefits, unemployment benefits, workman's compensation, help from relative (include child payments and alimony), and so on.”. Ten income brackets were provided as outcomes: <$5,000; $5,000-11,999; $12,000-15,999; $16,000-24,999; $25,000-34,999; $35,000-49,999; $50,000-74,999; $75,000-99,999; $100,000-199,999; $200,000+. The most common income bracket was $100,000-199,999 (N = 2,548; 26% of sample). Whilst strictly ordinal, this variable was treated as a continuous fixed effect in the analysis, following the assumption that family income would be linearly related to quality of the child’s mental health.

### Parental Education

We applied a dominance criterion to education status of both parents, which takes the highest education level attained (of either parent) to be the education status of the household. If there was missing data on the highest education level attained for one parent, the household education status became the highest level attained by the other parent.

The 21 levels of education provided by the ABCD were simplified into 7 levels: (1) Incomplete Schooling (grades 1-12 without graduation); (2) High School Degree/GED; (3) Some College (without graduation); (4) Associate Degree (occupational/academic); (5) Bachelor Degree; (6) Master’s Degree; (7) Doctoral Degree/Professional School degree (e.g. MD). This variable was also treated as a continuous fixed effect in the analysis, in line with the assumption that parental educational level would be linearly related to the quality of the child’s mental health.

### **Single Parenthood.**

Single-parenthood was established by the question “do you have a partner?” to the primary respondent. Partner was specified as anyone who helps in raising the child or has helped for more than 2 years. This person had to be involved 40% or more of the child’s daily activities and could be a spouse, boyfriend/girlfriend, relative or friend of the parent.

# Family History of Mental Illness

The parent of every child was asked whether any blood relative of the child had ever experienced a range of psychological and behavioral conditions using the following conditions:

1. **Depression**: Has any blood relative of your child ever suffered from depression, that is, have they felt so low for a period of at least two weeks that they hardly ate or slept or couldn't work or do whatever they usually do?
2. **Problems with nerves**: Has any blood relative of your child ever had any other problems with their nerves, or had a nervous breakdown?
3. **Mania**: Has any blood relative of your child ever had a period of time when others were concerned because they suddenly became more active day and night and seemed not to need any sleep and talked much more than usual for them?
4. **Psychosis**: Has any blood relative of your child ever had a period lasting six months when they saw visions or heard voices or thought people were spying on them or plotting against them?
5. **Drug abuse:** Has any blood relative of your child ever had any problems due to drugs, such as: marital separation or divorce; laid off or fired from work; arrests or DUIs; Drugs harmed their health; in a drug treatment program; suspended or expelled from school 2 or more times; isolated self from family, caused arguments or were high a lot.
6. **Alcohol abuse:** Has any blood relative of your child ever had any problems due to alcohol, such as: marital separation or divorce; laid off or fired from work; arrests or DUIs; alcohol harmed their health; in an alcohol treatment program; suspended or expelled from school 2 or more times; isolated self from family, caused arguments or were drunk a lot.
7. **Antisocial behavior:** Has any blood relative of your child been the kind of person who never holds a job for long, or gets into fights, or gets into trouble with the police from time to time, or had any trouble with the law as a child or an adult?
8. **Suicidality**: Has any blood relative of your child ever attempted or committed suicide?

# Choosing the distribution of best fit

The R function fitdist() from the fitdistrplus package was used to fit CBCL scores (total & sub-scores) to both a gaussian and a gamma distribution. Fitting is done via maximum likelihood estimation and the fit parameters AIC & BIC help decide the distribution of best fit (in addition to residuals plots from fitted models). Lower AIC and BIC values indicate better fit. [Table S1](#TableS1.FitStatisticsForCbclOutcomesForA) shows AIC and BIC values are much lower for gamma distributions for all CBCL scores therefore we conclude that a gamma distribution fits the outcomes better than a gaussian.

Table S2.

Fit statistics for CBCL outcomes for a normal vs gamma distribution. Distribution of best fit highlighted in bold.

| Outcome | Distrib. | AIC | BIC |
| --- | --- | --- | --- |
| Total Problems | norm | 84227 | 84242 |
|  | **gamma** | **77379** | **77393** |
| Anxious-Depressive | norm | 49769 | 49783 |
|  | **gamma** | **42969** | **42983** |
| Withdrawn-Depressive | norm | 38400 | 38414 |
|  | **gamma** | **30556** | **30570** |
| Somatic Problems | norm | 41061 | 41075 |
|  | **gamma** | **34931** | **34945** |
| Social Problems | norm | 44069 | 44083 |
|  | **gamma** | **36731** | **36745** |
| Thought Problems | norm | 43288 | 43303 |
|  | **gamma** | **36369** | **36383** |
| Attention Problems | norm | 52322 | 52336 |
|  | **gamma** | **46092** | **46106** |
| Rule-breaking | norm | 40147 | 40162 |
|  | **gamma** | **32423** | **32437** |
| Aggression | norm | 56498 | 56513 |
|  | **gamma** | **47726** | **47741** |
| ASD scale | norm | 38479 | 38493 |
|  | **gamma** | **31747** | **31762** |

# Other (non-birth weight) predictors of CBCL

Full results of the model predicting CBCL total problems is provided below ([Table S2](#TableS2)). It shows that, by far the most reliable predictor of age 9-10 CBCL total problems was family mental health history. Recall that this variable was a count of all the types of mental health issue (e.g. depression, substance-abuse) in the extended family. For every additional mental health problem in the family, there is a predicted 2.62 increase in Total Problems score. The next most reliable predictors were being male and being from a lower income household. Being of Black, Asian or Hispanic race/ethnicity provided a protective effect in comparison to being White, while being from the “other” category was associated with increased problem scores.

Plotted effects of gestational age group on CBCL total problems and each sub-scale are provided in [Fig. S1](#FigureS1). Table 2 in the main text showed that the gestational age group with the highest CBCL total problem scores (in relation to those born full-term i.e. 39 weeks or more) was the early-term group (37-38 weeks). [Fig. S1](#FigureS1) shows that this early-term born group of children are characterized by particularly high scores on the anxious-depressed scale and to a lesser extent on the thought problems and aggressive behavior scales.

Table S3.

Effect Estimates for All Predictors of CBCL Total Problems. Not Including Adjustment for Gestational Age.

|  | M1 | | M2 | | M3 | |
| --- | --- | --- | --- | --- | --- | --- |
|  | Adjusted for sex | | M1 + Adjusted for socioeconomic factors & race/ethnicity | | M2 + Adjusted for race/ethnicity & family history of mental illness | |
|  | *B (SE)* | *t* | *B (SE)* | *t* | *B (SE)* | *t* |
| Birth Weight (kgs) | -0.43 (0.16) | -2.70** | -0.69 (0.18) | -3.90*** | -0.36 (0.15) | -2.35* |
| Sex (male) | 3.95 (0.19) | 21.08*** | 3.50 (0.20) | 17.71*** | 2.20 (0.17) | 13.09*** |
| Parent Income Bracket (1-10) |  |  | -0.74 (0.07) | -10.65*** | -0.42 (0.06) | -7.41*** |
| Parent Education Level (1-7) |  |  | -0.05 (0.09) | -0.61 | 0.17 (0.08) | 2.21* |
| Single-Parent Family |  |  | 2.03 (0.33) | 6.21*** | 0.09 (0.26) | 0.36 |
| Race/Ethnicity (ref. White) |  |  |  |  |  |  |
| Black |  |  | -3.29 (0.36) | -9.17*** | -1.46 (0.31) | -4.72*** |
| Asian |  |  | -4.24 (0.42) | -10.18*** | -1.33 (0.38) | -3.54*** |
| Hispanic |  |  | -1.51 (0.30) | -4.99*** | -0.85 (0.27) | -3.12** |
| Other |  |  | 1.46 (0.38) | 3.84*** | 1.09 (0.34) | 3.24** |
| Family Mental Health Issues (0-8) |  |  |  |  | 2.63 (0.06) | 42.96*** |
| *** *p* < 0.001 ***p* < 0.01 **p* < 0.05 | | | | | |  |

Fig. S1.

Effects of Gestational Age on CBCL Total Problems and all 9 Sub-Scales.


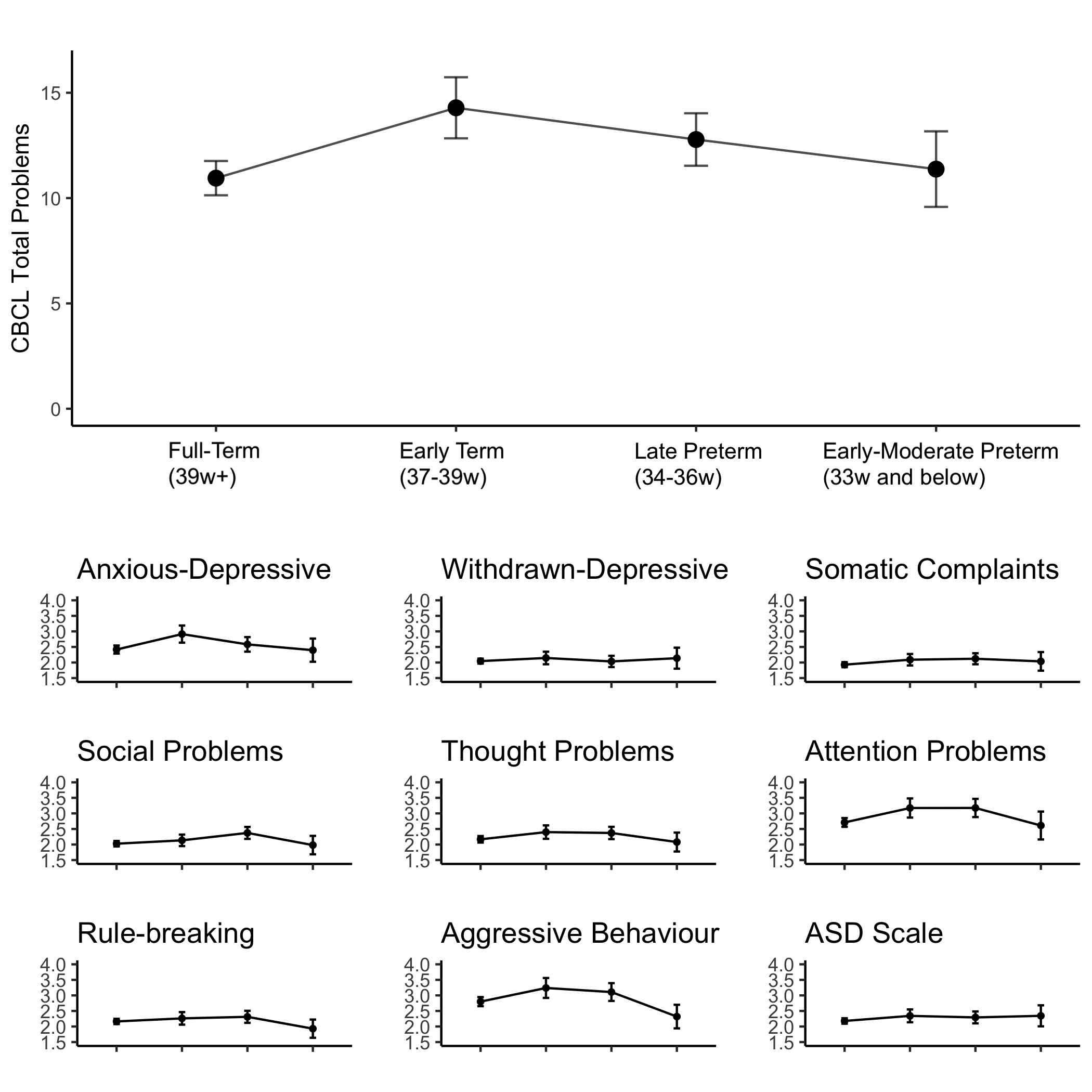


*Note*: Plots created using value estimates from M3 (i.e. adjusted for sex, socioeconomic factors, race/ethnicity and family history of mental illness). Estimates were also adjusted for birth weight.

# CBCL sub-score results

Table S4.

Linear Effect of Birth Weight on each CBCL Sub-Score (M3 estimates), Before and After adjustment for Gestational Age.

|  | **Unadjusted for gestational age**  (*N*=8,183) | | |  | **Adjusted for**  **gestational age**  (*N*=8,142) | | |
| --- | --- | --- | --- | --- | --- | --- | --- |
|  | ***β*** | ***SE*** | ***p*** |  | ***β*** | ***SE*** | ***p*** |
| Attention Problems | -0.21 | 0.04 | <0.001*** |  | -0.15 | 0.05 | 0.001*** |
| Somatic Problems | -0.09 | 0.03 | <0.001*** |  | -0.09 | 0.03 | 0.005*** |
| Social Problems | -0.04 | 0.03 | 0.14 |  | -0.004 | 0.03 | 0.89 |
| Aggression | -0.05 | 0.04 | 0.20 |  | -0.03 | 0.05 | 0.54 |
| Thought Problems | -0.04 | 0.03 | 0.15 |  | -0.03 | 0.03 | 0.44 |
| Rule-breaking | -0.01 | 0.03 | 0.72 |  | -0.004 | 0.04 | 0.90 |
| Withdrawn-Depressive | -0.03 | 0.03 | 0.40 |  | -0.02 | 0.04 | 0.56 |
| Anxious-Depressive | -0.07 | 0.04 | 0.05 |  | 0.04 | 0.04 | 0.32 |
| ASD scale | -0.05 | 0.03 | 0.11 |  | -0.02 | 0.03 | 0.59 |
| Uncorrected **p* < 0.05 ***p* < 0.01. Bonferroni Corrected ****p* < 0.006 | | | | | | | |

*Note:* These estimates are shown graphically in [Fig. 1](#Figure1). All estimates are corrected for sex, race/ethnicity, socio-economic factors and family history of mental illness (i.e. M3).

## Quadratic Effects of Birth Weight on CBCL Scales

There were no significant quadratic effects of birth weight on CBCL total problems (Table S5, 1st row). There was one significant quadratic effect of birth weight amongst CBCL sub-scores: aggressive behavior. Fig. S2 shows that aggressive behavior was rated highest for children of mean birth weight and comparatively lower for both low and high birth weight children. Despite this finding, interactions between birth weight and sex were only performed for linear representations of birth weight for simplicity and correspondence across all sub-scores.

Table S5.

Quadratic effects of birth weight on CBCL total score & sub-scores (M3, uncorrected for gestational age). Values represent beta and standard error.

|  | **M1** | **M2** | **M3** |
| --- | --- | --- | --- |
| Outcome | Adjusted for **sex** | Adjusted for sex, **socioeconomic** factors & **race/ethnicity** | Adjusted for sex, socioeconomic factors, race/ethnicity & **family history of mental illness** |
| Total Problems | 0.20 (0.16) | 0.31 (0.19) | 0.21 (0.16) |
|  | *p=0.21* | *p=0.10* | *p=0.20* |
| Anxious-Depressive | 0.008 (0.03) | -0.004 (0.04) | -0.007 (0.04) |
|  | *p=0.81* | *p=0.90* | *p=0.85* |
| Withdrawn-Depressive | 0.02 (0.03) | -0.01 (0.03) | -0.03 (0.03) |
|  | *p=0.55* | *p=0.65* | *p=0.41* |
| Somatic Complaints | 0.03 (0.02) | 0.03 (0.03) | 0.03 (0.03) |
|  | *p=0.21* | *p=0.25* | *p=0.36* |
| Social Problems | 0.04 (0.03) | 0.02 (0.03) | 0.006 (0.03) |
|  | *p=0.14* | *p=0.55* | *p=0.83* |
| Thought Problems | -0.004 (0.03) | -0.02 (0.03) | -0.04 (0.03) |
|  | *p=0.89* | *p=0.47* | *p=0.20* |
| Attention Problems | 0.007 (0.04) | -0.02 (0.04) | -0.05 (0.04) |
|  | *p=0.85* | *p=0.61* | *p=0.22* |
| Rule-breaking | <0.001 (0.03) | -0.01 (0.03) | -0.03 (0.03) |
|  | *p=0.99* | *p=0.76* | *p=0.37* |
| Aggressive Behavior | -0.07 (0.04) | -0.12 (0.04)** | -0.13 (0.04)** |
|  | *p=0.08* | *p=0.004* | *p=0.001* |
| ASD Scale | 0.05 (0.03) | 0.03 (0.03) | 0.03 (0.03) |
|  | *p=0.10* | *p=0.32* | *p=0.44* |
| **p* < 0.05 ***p*<0.01 ****p*<0.001 (uncorrected for multiple testing) | | | |

Fig. S2. Association between birth weight and aggressive behaviour (CBCL) significant quadratically (solid line; Table S5) but not significant linearly (hatched line; Table S4).
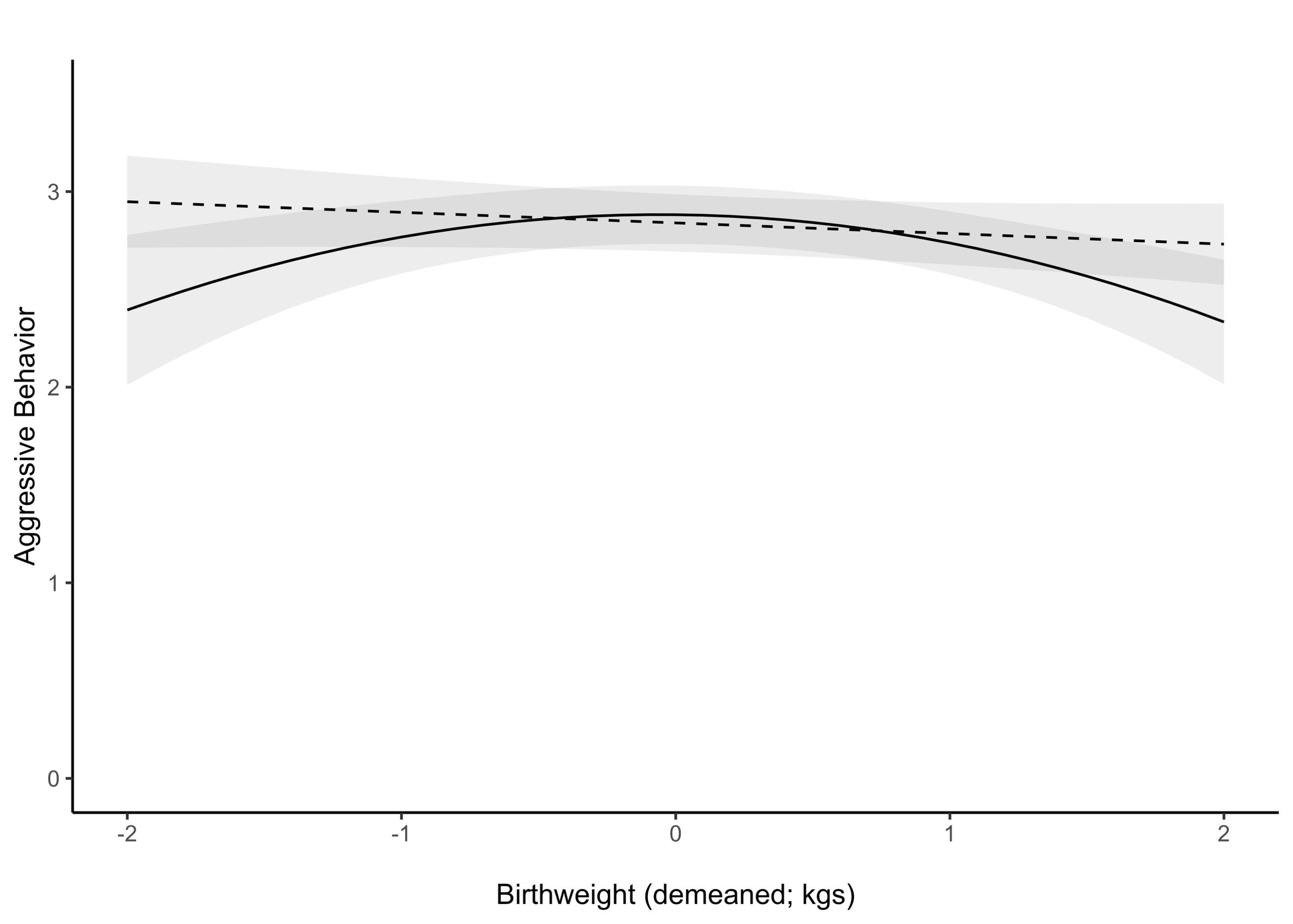


## Sensitivity Analyses

Several sensitivity analyses were run to explore the reliability of early-term born children (37-38 weeks’ gestation) having the highest CBCL total problem scores.

### 1. Equally Sized Gestational Age Groups

There were relatively few participants born at 33 weeks’ gestation or less (n=148) compared to those born in later gestational age groups (late preterm [n=408], early term [n=328], full term [n=8585]. This may have contributed to lack of linear association between decreasing gestational age and increasing CBCL total problems. We re-ran the analysis using equally sized gestation groups (n=140) for the 3 test groups i.e. early term, late preterm, moderate-early preterm. We randomly selected (without replacement) 1000 full-term born subjects for the reference group. We repeated the analyses (M1) 4 times using 4 different randomly selected sub-samples (seeds 1, 16, 42, 123). Fig. S3 below shows that despite equal group sizes, being born early-term still had the largest and most reliable effect on CBCL total problem scores across 4 seed values for random number generation.

Fig. S3. CBCL Total Problem Scores across Gestational Age Groups with Equal Numbers in each Group (reference group = full term births).


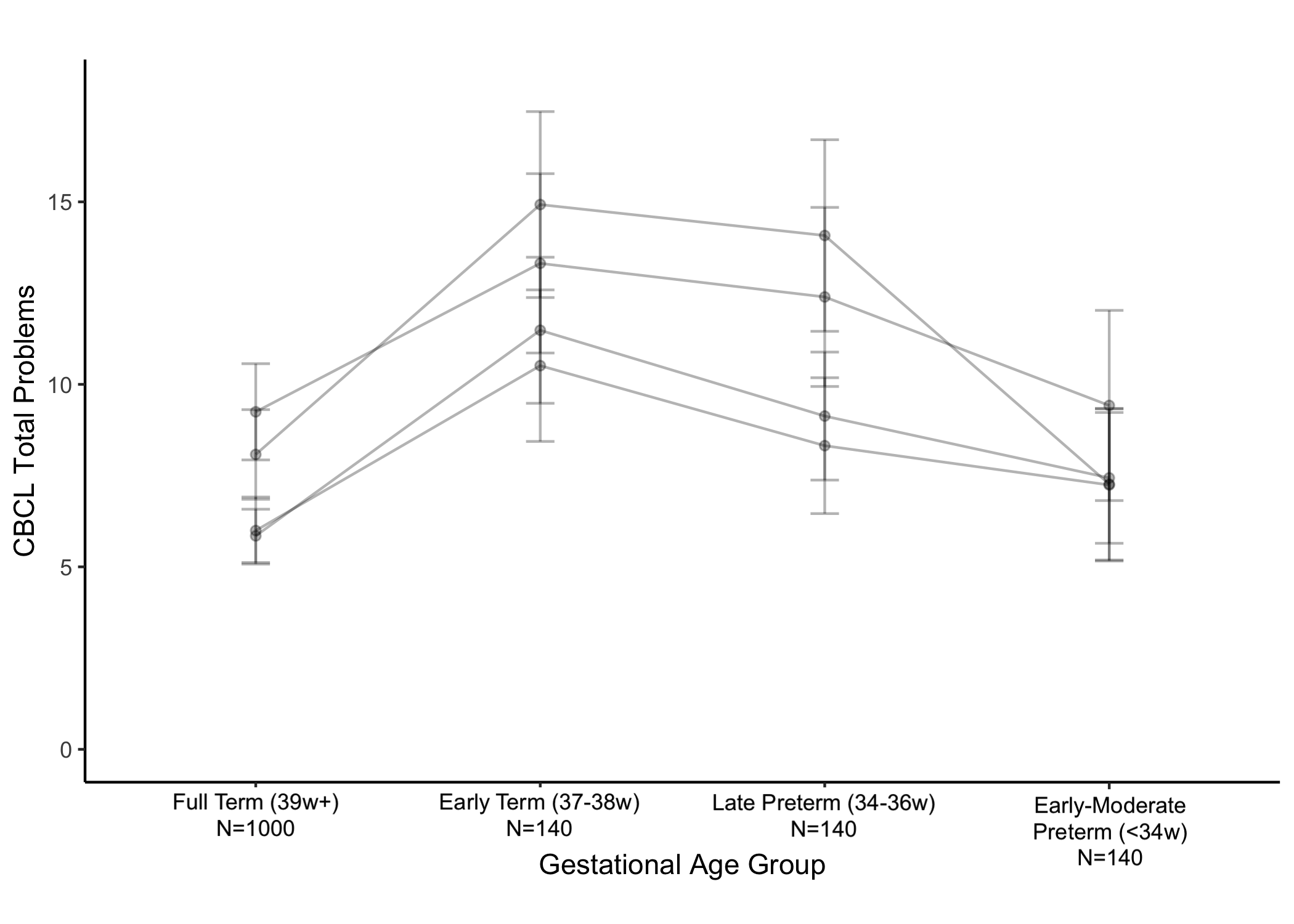
*Note*: The 4 lines refer to 4 randomly selected sub-samples using different random seeds (1, 16, 42 and 123). Plotted estimates were from M1 (fixed effects: birth weight, gestational age, sex).

### 2. Equal Proportions of Males and Females

Across all gestational age groups, there were more males than females, but the early term group had a higher proportion of males than all other groups (57% male; Table S6). For this reason, we ran the study with a restricted male sample, such that there were equal numbers of males and females within each gestational age group. We randomly removed males from the full-term, early-term, late preterm and early-moderate preterm groups respectively using random seeds of 1, 16, 42 and 123.

Table S6.

Original Proportions of Males and Females in each Gestational Age Group

| Gestational Age | Male (n=5,109) | | Female (n=4,624) | |
| --- | --- | --- | --- | --- |
|  | *n* | % | *n* | % |
| Full-term (39+ weeks) | 4,482 | 52 | 4,100 | 48 |
| Early-term (37-38weeks) | 188 | 57 | 140 | 43 |
| Late preterm (34-36 weeks) | 228 | 56 | 180 | 44 |
| Early-mod. preterm (33 weeks or less) | 78 | 53 | 70 | 47 |

There were 8,980 subjects in this sensitivity analysis (4,490 of each sex). Despite equal proportions of males and females, being born early-term still had the largest and most reliable effect on CBCL total problem scores across 4 seed values for random number generation (Fig. S4).

Fig. S4

CBCL Total Problem Scores across Gestational Age Groups with Equal Numbers of Males and Females.


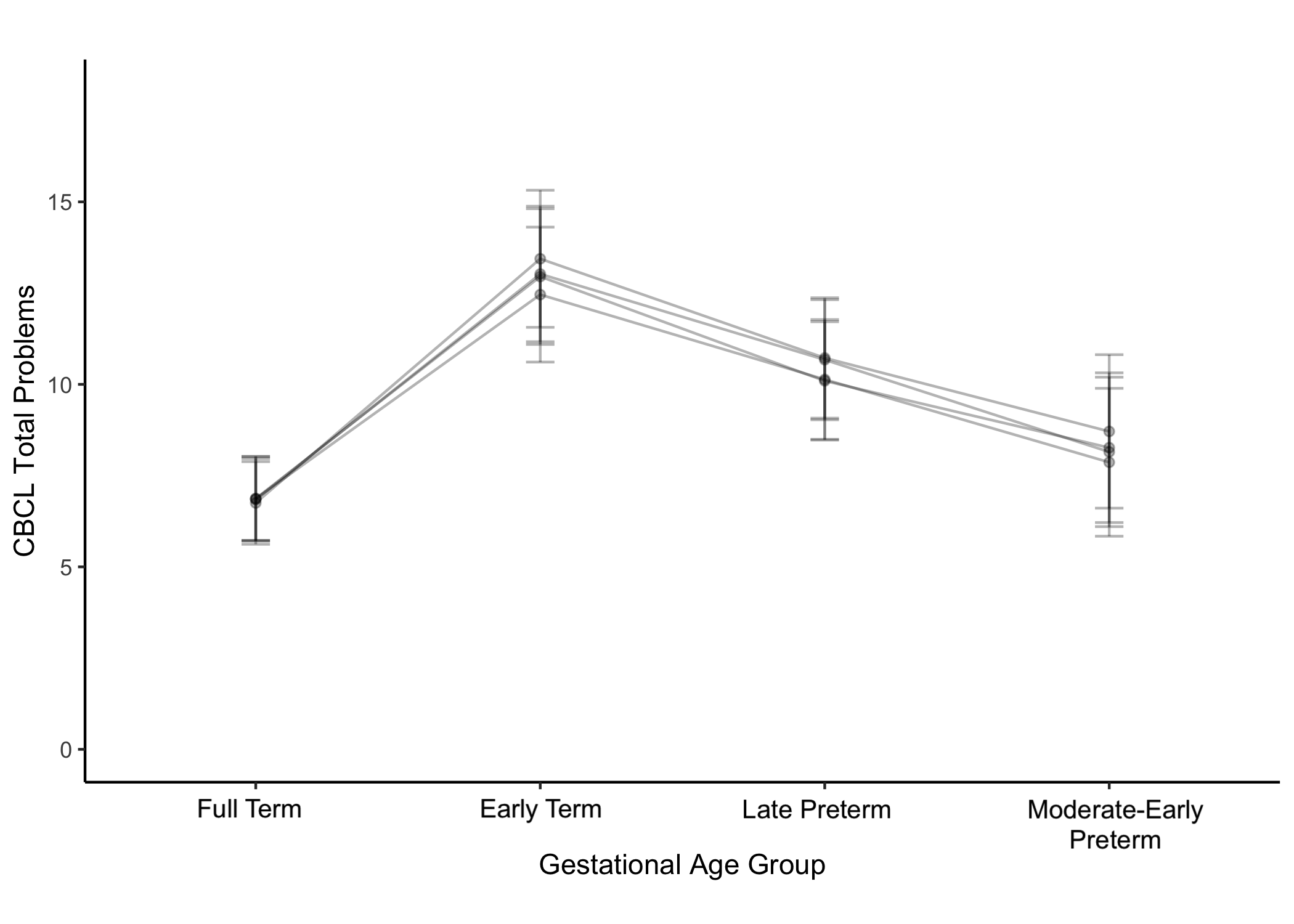


*Note*: The 4 lines refer to 4 randomly selected sub-samples with equal numbers of males and females chosen using different random seeds (1, 16, 42 and 123). Plotted estimates were from M1 (fixed effects: birth weight, gestational age, sex).

### 3. Excluding Outliers

Boxplots of CBCL total problem scores across each gestational age group (Fig. S5) show outliers in all groups but 3 particularly isolated outliers in the early-term group. We re-ran the main analyses without these 3 individuals.

Fig. S5

Boxplot for CBCL Total Problem Scores across Gestational Age Groups


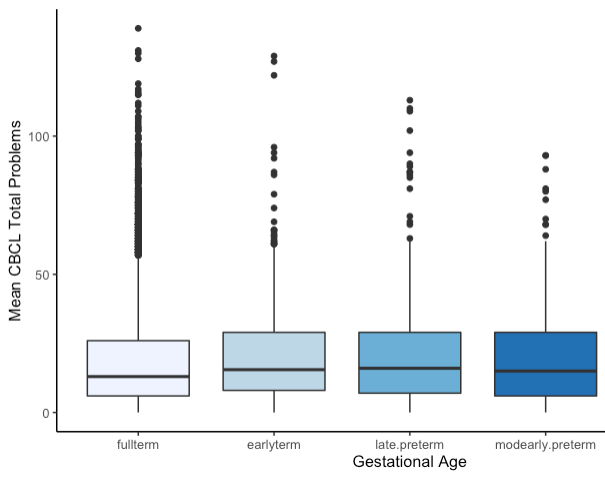


Full-Term

Early-Term

Late Preterm

Early-Mod. Preterm

No major change was observed in results. The same elevation in CBCL total problems amongst early-term born children remained (*β* = 5.96, *SE*=0.69, *t* = 8.60, *p* < .001) in comparison to term children born after 39 weeks’ gestation. Effect estimates were relatively unchanged for the other gestational groups of late preterm (*β* = 3.65, *SE* = .59, *t* = 6.21, *p* < .001) and early-moderate preterm (*β* = 1.10, *SE* = .84, *t* = 1.31, *p* = .19). Reported statistics are from M1 including adjustment for birth weight and sex (comparable to Table 2; column 1).

References

Achenbach, T., Rescorla, L., 2001. Manual for the ASEBA school-age forms & profiles: an integrated system of multi-informant assessment Burlington, VT: University of Vermont. Research Center for Children, Youth, & Families 1617.

Ooi, Y.P., Rescorla, L., Ang, R.P., Woo, B., Fung, D.S., 2011. Identification of autism spectrum disorders using the Child Behavior Checklist in Singapore. Journal of Autism and Developmental Disorders 41, 1147-1156.
